# What impact do posters have on academic knowledge transfer? A pilot survey on author attitudes and experiences

**DOI:** 10.1186/1472-6920-9-71

**Published:** 2009-12-08

**Authors:** Nicholas Rowe, Dragan Ilic

**Affiliations:** 1 School of Health & Social Care, Bournemouth University, Bournemouth, UK; 2 Monash Institute of Health Services Research, School of Public Health & Preventive Medicine, Monash University, Clayton, Australia

## Abstract

**Background:**

Research knowledge is commonly facilitated at conferences via oral presentations, poster presentations and workshops. Current literature exploring the efficacy of academic posters is however limited. The purpose of this initial study was to explore the perceptions of academic poster presentation, together with its benefits and limitations as an effective mechanism for academic knowledge transfer and contribute to the available academic data.

**Methods:**

A survey was distributed to 88 delegates who presented academic posters at two Releasing Research and Enterprise Potential conferences in June 2007 and June 2008 at Bournemouth University. This survey addressed attitude and opinion items, together with their general experiences of poster presentations. Descriptive statistics were performed on the responses.

**Results:**

A 39% return was achieved with the majority of respondents believing that posters are a good medium for transferring knowledge and a valid form of academic publication. Visual appeal was cited as more influential than subject content, with 94% agreeing that poster imagery is most likely to draw viewer's attention. Respondents also believed that posters must be accompanied by their author in order to effectively communicate the academic content.

**Conclusion:**

This pilot study is the first to explore perceptions of the academic poster as a medium for knowledge transfer. Given that academic posters rely heavily on visual appeal and direct author interaction, the medium requires greater flexibility in their design to promote effective knowledge transfer. This paper introduces the concept of the IT-based 'MediaPoster' so as to address the issues raised within published literature and subsequently enhance knowledge-transfer within the field of academic medicine.

## Background

The dissemination of new research knowledge, or knowledge transfer, has traditionally been facilitated by oral, poster and workshop presentations. Oral presentations generally consist of detailed information presented in a didactic format. Poster presentations provide the viewer with a 'snap shot' of the topic of interest. [1-5] Workshops are increasingly being included in conferences to facilitate the active learning of skills and methodologies.

The static nature of the poster presentation may not be perceived as promoting an environment that is conducive to 'active learning', although it still meets the expressed professional demand for a constant 'snap-shot' overview. In itself, active learning consists of strategies that encourage the learner to engage in activities including the analysis, synthesis and evaluation of information. [6] Support of this methodology is present in literature, [7] and links closely with the practice learning approaches commonly employed within medicine and healthcare.

Poster presentations may stand alone or be accompanied by a short presentation (less than five minutes in duration) by the presenter. [1] Unsupported poster presentations may elicit a degree of active learning, in that the audience must engage with the poster to read, synthesise and analyse the information presented. [1,2] However, this has limited potential and increases the reliance on visual themes to attract viewer attention. Combining a short presentation or author presence with the poster presentation can facilitate discussion between the presenter and audience. [1,8] This process may be altogether more engaging and another means by which the poster presentation can promote active learning.

The 'traditional' poster presentation aims to present information in a succinct manner. In an effort to deliver a depth of information that supports their work, the final result is often a congested poster in which the key messages are either difficult to find or lost. [1,2,4,9] Some posters may have tremendous visual appeal, but lack content. [1] This has led to a perception that the medium is lacking in terms of depth and interactivity and as such, not perhaps given the appropriate level of academic appreciation that it deserves. [2,9,10]

Given that posters are primarily acknowledged to be a visual medium, 'traditional' poster presentations are not well equipped to accommodate alternative learning styles. Whilst an audience may consist of those who best learn when reading information, a poster also needs to provide clear navigation in either vertical (top to bottom or vica versa) or horizontal (left to right or vica versa) planes in order to provide a sequential logic. [3,4,11] Given its passive nature; if not accompanied by a short presentation which can help with aural and verbal learning exchange, the content of the 'traditional' academic poster may only reach a limited proportion of its intended audience. [12]

A growing body of literature serves to examine the overall effectiveness of knowledge transfer within the medical and healthcare fields, although comparatively little is published exploring the poster medium. [13-15] The purpose of this study was to explore the perceptions of the academic poster presentation, together with its benefits and limitations as a mode for academic knowledge transfer.

## Methods

Two academic conferences were held at Bournemouth University in June 2007 and June 2008. These were regional based conferences with the majority of attendees from the United Kingdom (UK). Following on from programmes which aimed to release the research and enterprise potential of academics from a broad range of disciplines, the conference events centred upon a range of accompanied poster presentations, at which delegates sought to disseminate independent work that had been generated during their programme participation. These meetings were selected as representative of a multi-disciplinary conference which utilised poster presentations within its structure. Delegates had undergone a year-long development programme to further their potential for research participation within their academic fields. Delegates at both conferences spent three 30 minute sessions to view the posters and interact with the respective authors. Delegates also had the option of viewing the posters over lunch.

An online survey was designed to assess the attitudes, opinions and experiences of conference delegates on academic poster presentations. Outcomes were measured on a 5-point Likert scale (1 = strongly disagree, 2 = disagree, 3 = neither agree/disagree, 4 = agree, 5 = strongly disagree). Four questions were also asked to identify participants' previous observations of academic posters. Participants were asked to state whether they recalled a specific poster from the conference attended, whether they discussed the poster with the author and whether there was any follow-up to the discussion. All of these outcomes were answered yes, no or don't recall. Participants were also asked to identify whether they best remembered the image or content of the poster. Open-ended questions were also available for participants to make any qualitative responses.

A total of 87 posters were presented at the two meetings. Authors from each poster were sent an email requesting them to participate in an online survey, incorporating the above mentioned survey, as part of this study. The survey incorporated a number of features that have been illustrated to increase participant response rate including; online delivery via a short questionnaire, an offer of overall survey results, the use of textual representation of response categories, a deadline and reminder by when to submit the survey and use of a novel topic. [16]

Descriptive statistics (mean, standard deviation, and percentages) were determined for each statement. Modified thematic analysis was used to interpret qualitative responses from the open-ended questions. [17] Ethics approval of the research was obtained from the organisers of the programmes concerned and in-line with IRB institutional policy (including the Bournemouth University Research Committee and the School of Health & Social Care Research Committee).

## Results

A total of 34 surveys from conference participants were returned, for a response rate of 39%. All respondents were academics attending two research & enterprise developmental conferences in successive years. The conferences were multi-disciplinary although the professional and academic level of attendee was representative of that to be seen at specific healthcare conferences. The majority of respondents were female (67%). Respondents were aged predominantly below 49 years (72%). Approximately half of the respondents had previously participated in some form of formal training or workshop on how to develop an academic poster presentation.

Respondent attitudes and opinions regarding academic posters are summarised in Table 1. Overall 62% of participants agreed, or strongly agreed, with the statement that posters are a good medium for knowledge transfer in the academic environment. Half of the participants believed that posters are a valid format of academic publication, however only 32% agreed that the medium permits the author to convey a suitable depth of subject information to the viewer. Participants were divided on their attitude on whether posters allow the author to present considered academic debate on the topic, with 41% agreeing with the statement and 53% disagreeing.

**Table 1:** Responses to survey (attitudes and opinions)

Questions	Mean (SD)	No. (%) strongly disagree	No. (%) disagree	No. (%) neither agree or disagree	No. (%) agree	No. (%) strongly agree
**Academic communication**						
Academic Conference Posters (Posters) are commonly used with my professional field	3.53 (1.10)	2 (5.9)	4 (11.8)	8 (23.5)	14 (41.2)	6 (17.6)
Posters are a good medium for knowledge transfer	3.68 (0.98)	0 (0)	5 (14.7)	8 (23.5)	14 (41.2)	7 (20.6)
Posters are generally considered a valid means of academic publication	3.09 (1.08)	4 (11.8)	6 (17.6)	7 (20.6)	17 (50)	0 (0)
Posters convey a suitable depth of subject information to the viewer	2.94 (0.85)	0 (0)	13 (38.2)	10 (29.4)	11 (32.4)	0 (0)
Posters allow the author to present a considered academic debate	2.94 (1.07)	0 (0)	18 (52.9)	2 (5.9)	12 (35.3)	2 (5.9)
**Communication**						
The information provided by Posters is generally sufficient to stand alone	2.59 (0.99)	2 (5.9)	18 (52.9)	8 (23.5)	4 (11.8)	2 (5.9)
Conference delegates often discuss a Posters content with others	3.44 (1.24)	3 (9.4)	6 (18.8)	2 (6.3)	16 (50)	5 (15.6)
Authors are normally attendant to expand upon issues raised within their Posters	3.79 (0.85)	0 (0)	4 (11.8)	4 (11.8)	21 (61.8)	5 (14.7)
Posters effectively convey a lasting message to the viewer	3.50 (0.83)	0 (0)	3 (8.8)	15 (44.1)	12 (35.3)	4 (11.8)
Posters provide benefit to viewers, after conference proceedings have concluded	3.44 (0.96)	1 (2.9)	4 (11.8)	12 (35.3)	13 (38.2)	4 (11.8)
**Art versus knowledge transfer**						
The visual appeal of Posters is more influential on audience appreciation than text	3.91 (0.62)	0 (0)	1 (2.9)	5 (14.7)	24 (70.6)	4 (11.8)
Catching viewers attention is reliant upon imagery and composition	4.21 (0.54)	0 (0)	0 (0)	2 (5.9)	23 (67.6)	9 (26.5)
Posters alone, represent the authors artistic capability more than their scholarly content	2.68 (1.07)	3 (8.8)	15 (44.1)	8 (23.5)	6 (17.6)	2 (5.9)
Poster compilations accurately reflect the work that has gone into the subject issue	3.06 (0.74)	0 (0)	8 (23.5)	16 (47.1)	10 (29.4)	0 (0)

* Items were scored on a 5-point scale, where 1 = strongly disagree, 3 = neither agree or disagree, 5 = strongly agree

The majority of participants believed that the visual aspects of an academic posters was more appealing to viewers that the text and subject content. Almost all of the participants (94%) believed that the imagery and composition of the poster (e.g. colours, figures) was the main factor in catching the viewer's attention. However, over half of respondents did not believe that the author's artistic ability disguised the scholarly content offered in the poster (53%).

Participants agreed that the information provided by posters needed to be supplemented by some form of oral presentation, or author presence, to further communicate the content. Approximately two-thirds of participants believed that conference delegates discussed poster presentations during conferences, with 76% believing that authors should be present with their poster. Participants were divided in their belief as to whether posters effectively communicated a lasting message to the viewer, with 47% agreeing or strongly agreeing with the statement, whilst 44% were uncommitted. A similar trend was also observed when identifying the long term impact of knowledge translation post conference.

Participants were also asked about their own experiences with constructing and presenting their poster at the conferences, with the majority of participants' answers endorsing their previous responses on the various topics (Table 2). Half of the participants believed that their poster was an effective medium for knowledge transfer, with 59% judging that it sufficiently attracted the attention of conference delegates. Almost half of the respondents (47%) acknowledged some formal training in designing academic posters, and the majority (70%) found it easy to construct a poster with balanced text and graphical content. Despite 59% of participants stating that knowledge transfer is not achieved when posters are 'stand alone', half of the respondents believed that their poster would have worked as a stand-alone presentation at the recent conference.

**Table 2:** Responses to survey (experiences with poster presentations)

Questions	Mean (SD)	No. (%) strongly disagree	No. (%) disagree	No. (%) neither agree or disagree	No. (%) agree	No. (%) strongly agree
My poster conveyed all the detail needed for viewers to understand the issues involved	3.21 (1.11)	2 (5.9)	9 (26.5)	6 (17.6)	14 (41.2)	3 (8.8)
My poster attracted the attention of a range of other delegates	3.44 (1.16)	3 (8.8)	4 (11.8)	7 (20.6)	15 (44.1)	5 (14.7)
My poster was an effective medium of knowledge transfer	3.38 (1.05)	2 (5.9)	4 (11.8)	11 (32.4)	13 (38.2)	4 (11.8)
I found it easy to balance words & imagery within my Poster	3.59 (0.99)	0 (0)	8 (23.5)	2 (5.9)	20 (58.8)	4 (11.8)
I think my poster provided a fair representation of my work	3.68 (0.88)	0 (0)	5 (14.7)	5 (14.7)	20 (58.8)	4 (11.8)
My poster would have worked as a stand-alone presentation	3.29 (1.29)	3 (8.8)	8 (23.5)	6 (17.6)	10 (29.4)	7 (20.6)

Items were scored on a 5-point scale, where 1 = strongly disagree, 3 = neither agree or disagree, 5 = strongly agree

Several open-ended questions elicited qualitative responses from participants. A total of 88% of participants recalled a specific poster from the event that they attended.

"They (posters) are a very useful way to include more (academic content) into a conference. New researchers especially can bring their work to a wider audience prior to the paper presentations which they will do later on in their academic careers."

Over two-thirds of participants (73%) remembered the imagery of the poster more clearly, rather than academic content.

"Posters appeal to me due to their visual component, but there are too many factors, both technical and behavioural, that restrict their value. They make a very efficient use of our time at external events as an ice-breaker for beginning discussions and establishing networks with colleagues."

Although 73% of respondents discussed the poster with the author, less than a third (30%) followed up on their discussion after the event. From an author's perspective:

"The poster medium is an excellent discipline for crystallizing your views on a topic. However unless you get a chance to discuss the poster with other delegates it can feel like you are having a discourse with yourself."

## Discussion

This pilot study is the first to evaluate the perception of the academic poster as a medium for knowledge transfer. The majority of literature currently published on the subject has concentrated on only discussing the benefits and limitations of the poster as an academic tool, and the generalised aspects of poster construction. The findings from this pilot study have identified that academic posters are perceived as a good medium for transferring knowledge and also a valid form of academic publication. It also identified that the visual appeal of posters is more influential than subject content, with poster imagery the most likely feature to draw viewers' attention to the poster and subsequently engage them. It is also evident that traditional academic posters must be accompanied by the author in order to effectively communicate the subject detail.

The characteristics that aim to promote knowledge transfer within 'traditional' poster presentation are, paradoxically, also features which may limit its effectiveness. As seen in our study, and supported by others, viewers may place more emphasis on 'first impressions' and characteristics such as presentation rather than scientific merit, a succinct message and originality. [18] Posters are designed to give a visual representation of an issue that firstly attracts attention, and then conveys an intended message. Therefore, much of the poster's success as an educational tool relies upon the design of the poster. Design layout, including colour schemes, framing of information and readability all influence how effectively the key information may be conveyed to the reader; sometimes at detriment to the overall scientific message that is presented. [1,3,4,11,18]

### Advances beyond paper

The digital interactive poster presentation (DIPP) was a new system proposed for organising poster sessions in 2001, and employed at the 14th Meeting of the European Association of Cardio-thoracic Surgery. [12] It aimed to overcome the limitations of 'traditional' posters by creating an interactive presentation which would encourage greater discussion between the audience and presenter. [12] The DIPP permitted presenters to project their poster on a large screen and magnify pre-selected sections (e.g. figures, text, tables) whilst providing a two minute summary of their poster. Whilst this contributed to the special quality of the presentation and ensured author participation; the enhanced illustration of imagery and data did not extend beyond that represented on the poster itself. An evaluation of the DIPP system demonstrated support towards a more interactive poster presentation system by both audience and presenters. [12]

### 'MediaPoster' - a practical development of the academic poster

In developing the 'MediaPoster' concept, we have looked to enable the combined evolution of the DIPP principle and its traditional forbear. [19] The 'MediaPoster' aims to combine information technology (IT) with a 'traditional' poster appearance, thus retaining the static image and at the same time releasing the full interactive potential of the medium. [19] 'MediaPoster' is presented from a laptop base onto interactive LCD or whiteboard screens, or even as a standard interactive computer screen display. It allows any individual section of a digital poster (full or screen size) to hold embedded links to supporting and additional information. Viewers can select an area of interest on the poster surface and access a full range of linked documents and imagery which open in a dedicated viewing area at the side of the screen (Additional file 1). The original poster image and information remains unchanged and in full view for others to see. All supporting documents are 'live' and allow open access to tables, PDF and other common publishing formats, which open in an enhanced view to aid readability. Authors may enable access to as much or as little supporting data and material as is appropriate, so assigning their own academic 'depth' to the medium. Restraints are only relative to the technological capability of the hardware system employed.

The limitations to this pilot study are acknowledged. Despite the use of established methods to increase participant response rate to our electronic survey, our response rate was 39%. Future follow-up surveys aim to incorporate additional measures, including personalising invitations, follow-up emails and incentives, in an attempt to increase response rate. [16] It is further recognised that due to the low response rate, the results of this study are limited in their generalisability. Participants in this study had undergone a year-long development programme specifically designed to better their research skills. This study represents attitudes and experiences of this selected group of academics, which may limit the findings widespread applicability. However, given that this is the first study to explore the perceptions, attitudes and experiences of the academic poster, the pilot nature of the data yields sufficient information to validate the construction of a larger survey instrument. It is anticipated that this instrument will be disseminated at several upcoming conferences across a variety of academic and medical disciplines.

## Conclusion

Poster presentations are a valid form of transferring academic knowledge. However, greater flexibility in their design and dissemination is required. 'MediaPoster' provides an opportunity to deliver a genuine depth of information, which is amendable to suit a wide variety of academic, professional and commercial disciplines. It accounts for a full range of learning styles by use of interactive delivery, and so promotes a genuine forum of active learning. Further research is however required and an interventional study is currently being devised on which to evaluate the effectiveness of the 'MediaPoster'.

## Competing interests

The authors declare that they have no competing interests.

## Authors' contributions

NR conceived the design of the study, collected and analysed the data, and drafted the manuscript. DI analysed the data and drafted the manuscript. Both authors read and approved the final manuscript.

## Author information

NR is a lecturer at the School of Health & Social Care, Bournemouth University. DI is a senior lecturer in Evidence Based Practice at the School of Public Health & Preventive Medicine, Monash University.

## Pre-publication history

The pre-publication history for this paper can be accessed here:

http://www.biomedcentral.com/1472-6920/9/71/prepub

## Supplementary Material

Additional file 1 **MediaPoster**. A graphical representation of the 'MediaPoster'. Click here for file
