# Supplementary figures and images for: What impact do posters have on academic knowledge transfer? A pilot survey on author attitudes and experiences
Source: BMC Med Educ. 2009 Dec 8;9:71. doi: 10.1186/1472-6920-9-71 (PMC2795740; doi:10.1186/1472-6920-9-71)

**Additional files**

Additional file 1: Graphical representation of the ‘MediaPoster’


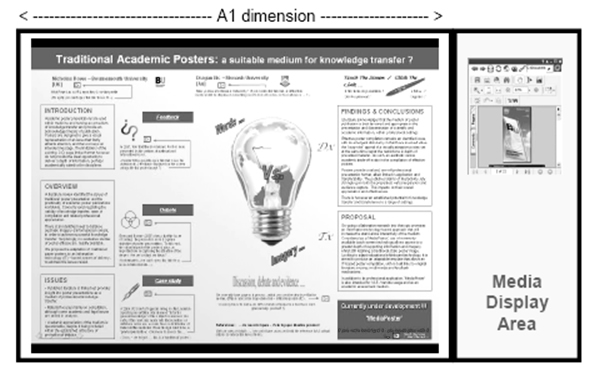

Supplement: Additional file 1 — MediaPoster. A graphical representation of the 'MediaPoster'. [file 1472-6920-9-71-S1.DOC]
